# Effects of Flywheel vs. Free-Weight Squats and Split Squats on Jumping Performance and Change of Direction Speed in Soccer Players

**DOI:** 10.3390/sports11070124

**Published:** 2023-06-23

**Authors:** Jakub Jarosz, Paulina Królikowska, Patryk Matykiewicz, Piotr Aschenbrenner, Paulina Ewertowska, Michał Krzysztofik

**Affiliations:** 1 Institute of Sport Sciences, The Jerzy Kukuczka Academy of Physical Education, 40-065 Katowice, Poland; j.jarosz@awf.katowice.pl (J.J.); p.krolikowska@awf.katowice.pl (P.K.); p.matykiewicz@awf.katowice.pl (P.M.); 2 Department of Biomechanics and Sports Engineering, Gdansk University of Physical Education and Sport, 80-336 Gdansk, Poland; piotr.aschenbrenner@awf.gda.pl; 3 Division of Clinical Physiotherapy, Faculty of Physical Education, Gdansk University of Physical Education and Sport, 80-336 Gdańsk, Poland; 4 Department of Sport Games, Faculty of Physical Education and Sport, Charles University in Prague, 110 00 Prague, Czech Republic

**Keywords:** resistance training, inertia training, eccentric, split squat, lateral squat, unilateral training

## Abstract

The objective of this study was to compare (i) The effects of a flywheel and free-weight resistance training program; and (ii) The effects of performing lateral and frontal split squats as part of a flywheel-resistance training program on jumping performance, the 5–0–5 change of direction test time, and the one-repetition maximum (1RM) back squat in soccer players. Twenty-four male amateur soccer players participated in this study and were randomly and equally assigned to one of three different test groups: forward split-squat group (FSQ); lateral split-squat group (LSQ); and free-weight training group (TRAD). Athletes in the FSQ group performed a squat and a forward split squat on a flywheel device, while those in the LSQ group performed a squat and a lateral split squat (instead of a forward split squat) on a flywheel device. Each training lasted 4 weeks. The main finding was that all training groups, such as TRAD, FSQ, and LSQ, significantly improved broad jump length (*p* = 0.001; effect size [ES] = 0.36), 5–0–5 COD time with a turn on the dominant limb (*p* = 0.038; ES = 0.49), and 1RM back squat (*p* = 0.001; ES = 0.4). In turn, both flywheel-resistance training groups (FSQ and LSQ) significantly improved their counter-movement jump height (*p* = 0.001; ES = 0.8 and *p* = 0.002; ES = 0.58; respectively) with no effect in the TRAD (*p* = 0.676; ES = 0.07) training group. Both free-weight and flywheel-resistance training lasting 4 weeks performed in-season contributed to significant improvement in 1RM back squat, broad jump performance, and 5–0–5 change of direction testing time, while flywheel-resistance training might be superior in counter-movement jump height enhancement in soccer players. Moreover, the manner in which split squats were performed was not a factor influencing the obtained results.

## 1. Introduction

The use of eccentric training has been of great interest to practitioners and researchers in recent years due to specific and quick adaptations to eccentric muscle actions [1,2,3]. Some physiological responses observed after eccentric muscle actions contribute to a new stimulus within the neuromuscular system [2,4,5]. Unique structural adaptations in muscle-tendon units evoke changes toward a faster, more explosive muscle phenotype [6,7,8]. Therefore, chronic eccentric resistance training has, in some studies, induced greater enhancements in muscle strength [9], power output [10,11], and stretch-shortening cycle function [12] compared with traditional resistance training alone.

Since, nowadays, soccer players must execute a variety of explosive movements, such as accelerations, jumps, sprints, and changes of direction (COD) [13], it seems crucial to identify and optimize training interventions that enhance power output. One training solution emphasizing eccentric actions in resistance training that is widely studied is the flywheel inertial training [14,15]. Flywheel devices use inertial resistance that results from the unwinding and rewinding of the flywheel’s cable. During the concentric phase of the exercise, the athlete extends the cable, leading to flywheel rotation and, as a result, creating inertial torque. The cable is then rewound, forcing the eccentric muscle action. Hence, the intensity of the eccentric phase depends on the effort put into the concentric phase: the greater the concentric acceleration, the greater the demands during the eccentric phase.

According to a recent systematic review conducted by Allen et al. [16], various interventions involving flywheel training have been shown to effectively enhance the strength, power, jumping, and change-of-direction abilities in male soccer players across different skill levels. However, there is limited evidence to unequivocally decide whether flywheel or free-weight resistance training interventions are better at improving the physical capacities of soccer players [17,18]. For instance, Coratella et al. [17] observed similar improvements in the one-repetition maximum (1RM) back squat and counter-movement jump (CMJ) height after flywheel and barbell squats. In turn, Sagelv et al. [18] reported significant improvements in 10-m sprint time and CMJ height after both the flywheel and free-weight squats. However, the 1RM back squat enhancement was significantly greater following free-weight squats. Yet none of these studies evaluated COD performance or assessed the effectiveness of unilateral exercises as part of flywheel training. This is especially important considering that the majority of on-field movements require players to produce force unilaterally and multi-directionally [19]. Considering the principle of specificity, unilateral exercises in different planes of motion (i.e., sagittal and frontal) should be considered during the design of a resistance-training program. Regarding that, to the best of the authors’ knowledge, there is a lack of studies that compared the effectiveness of lateral and frontal split squats as part of a flywheel resistance training on athletic performance in soccer players. Nevertheless, studies by Gonzalo–Skok et al. [20] and Raya–González et al. [21] showed substantial enhancements in CMJ height following flywheel lateral squats. In addition, Raya–González et al. [21] reported significantly greater improvements in COD (with a 90-degree turn) compared to soccer training, but not in the 10-m, 20-m, and 30-m sprint times. This may suggest that exercises performed in the frontal plane may contribute to improving COD but not linear sprint performance.

Therefore, the objective of this study was to compare (i) The effects of a flywheel and free-weight resistance training program; and (ii) The effects of performing lateral and frontal split squats as part of a flywheel resistance training program on jumping performance, the 5–0–5 COD test time, and the 1RM back squat in soccer players. It was hypothesized that 4 weeks of free-weight and flywheel resistance training would contribute to a significant improvement in all analyzed variables. However, the magnitude of improvements will vary depending on the training group, with lateral split squats enhancing COD performance the most, while frontal split squats improve jumping performance to the highest degree and free-weight training enhances the 1RM back squat.

## 2. Materials and Methods

### 2.1. Participants

Twenty-four male amateur soccer players participated in this study. They were randomly and equally assigned to one of three different test groups: forward-split squat group (FSQ) (*n* = 8; 19 ± 1 years; 177 ± 6 cm; 66 ± 5 kg; soccer training experience: 6 ± 1 years); lateral-split squat group (LSQ) (*n* = 8; 20 ± 1 years; 176 ± 10 cm; 70 ± 11 kg; soccer training experience: 6 ± 2 years) and free-weight training group (*n* = 8; 20 ± 1 years; 173 ± 7 cm; 64 ± 8 kg; soccer training experience: 6 ± 1 years). The criteria for inclusion were as follows: absence of any significant lower-limb injuries, such as tendon or muscle tears, within the two years preceding the study; a minimum of five years of training and competing experience; and consistent participation in resistance training for at least two years prior to the study. While all athletes reported having two years of experience with resistance training, they were not acquainted with the use of flywheels in resistance training.

All athletes maintained a consistent training regimen on the field five days per week, including the match, throughout the study and were involved in a training program of the current study two days per week. In the study, athletes were instructed to maintain their sleep hygiene and dietary habits, as well as abstain from utilizing stimulants. Tests were scheduled at the same time of day (15:00–17:00) for all testing and training sessions to mitigate the effects of the circadian rhythm. Additionally, they were asked not to engage in any additional resistance exercises 48 h before testing to minimize fatigue. The athletes were informed about the benefits and potential risks of the study prior to its commencement and provided written consent to participate. However, neither the athletes nor the coach who supervised the training were informed about the potential results. The study protocol was approved by the Bioethics Committee for Scientific Research at the Academy of Physical Education in Katowice, Poland (3/2021) and conducted in accordance with the ethical standards of the Declaration of Helsinki 2013.

### 2.2. Familiarization Session

Each session began with the same warm-up procedures, which consisted of 5-min cycling and a set of five repetitions of the following exercises: backward lunge + reach, leg cradle, knee hug, inverted hamstring with knee drive, drop lunge, and the world’s greatest stretch. Then, the athletes performed movement integration with one set of six repetitions: linear march, linear skip, next-neural activation by one set of 5 s of running in place, and base rotations. At the end of the warm-up, the athletes performed one set of 10 repetitions of bodyweight exercises: squats, forward and lateral split squats, and four repetitions of CMJ, SJ, and lateral jumps.

Before the training intervention, all athletes completed two familiarization sessions, including the COD and jumping tests in the first session and back squat 1RM in the second session a week later. Both sessions were followed by two sets of 10 repetitions of exercises performed on a flywheel device that was later used in the training programs, including the bilateral squat, forward split squat, and lateral split squat. All tests were repeated a week after completing the training program in the same order.

### 2.3. Training Program

The athletes were randomly divided into three groups: TRAD (without flywheel resistance training), FSQ, and LSQ. Athletes in each group performed resistance training twice a week with a 72-h recovery period in between. The training program consisted of the following exercises: barbell back squats, bench presses, forward split squats, pulldowns, barbell hip thrusts, and pall of press with a resistance band. However, the athletes in the FSQ group performed a squat and a forward split squat on a flywheel device, while those in the LSQ group performed a squat and a lateral split squat (instead of a forward split squat) on a flywheel device. Each training lasted 4 weeks. All exercises were performed with approximately 3-min rest intervals between sets, as well as 45-s rest intervals between exercises with 60% 1RM for three sets of 8–10 repetitions. Moderate intensity was selected to provide the optimal load for maximal power production [22]. The inertia load used was 0.10 kg/m^2^ (5 kg) for a back squat and 0.05 kg/m^2^ (3.52 kg) for split squats. Throughout all training sessions, to achieve eccentric overload, all participants were consistently motivated to perform the concentric phase with maximum speed while delaying the deceleration phase until the later part of the eccentric action. Similarly, during the traditional resistance exercises, the eccentric phase was controlled by a metronome to be performed in approximately 2 s.

### 2.4. Broad Jump Testing

Every athlete was positioning themselves on the starting line, aligning their legs parallel and placing their feet shoulder width apart. Next, they were given instructions to flex their knees (choosing the depth of flexion themselves) and position their arms behind their body. Subsequently, they generated a forceful drive by extending their legs, propelling their arms forward, and executing a maximal jump for distance. The measurement of the jump distance, in centimeters, was performed by the same individual who is a certified strength and conditioning coach. Athletes performed three attempts of the broad jump with 1-min rest intervals in between jumps. The best attempt was recorded for further analysis.

### 2.5. Change of Direction Performance Test

The established methodology for the 505-agility test, as outlined in existing literature [23], was employed. To commence the test, athletes positioned their preferred foot directly in front of the starting line. Upon the signal, the athletes sprinted through a timing gate towards a marked turning line on the floor. Depending on the trial, athletes were instructed to place either their left or right foot on or behind the turning line before swiftly sprinting back through the gate. Two trials were conducted for turns executed on both the dominant and non-dominant sides. The order of the trials was randomized among the participants, with a recovery period of 2 min provided between each trial. A Swift timing gate (Swift Performance, Wacol, Queensland, Australia) was used in the study. The time for each run was recorded to the nearest 0.01 s. The best attempt was preserved for further analysis.

### 2.6. Counter-Movement Jump Testing

A bilateral CMJ without arm swing was performed on force plates (Forcedecks, Vald Performance, Newstead, Queensland, Australia) and their associated software. All athletes performed three CMJs with a 30-s rest interval between each attempt. Athletes performed the jumps starting in a standing position with their hands on their hips; they flexed their knees using a self-selected depth and then jumped as high as possible. Athletes were instructed to jump with both feet and knees fully extended (no leg tucking was allowed). The following variables were evaluated: jump height (based on the flight time measurement), relative peak power, relative peak force, peak velocity, time to take off, and counter-movement depth. The best attempt in terms of jump height was preserved for further analysis.

### 2.7. Test 1RM Back Squat

The ea test was performed using a linear transducer (Gym Aware Power Tool, Kinetic Performance Technologies, Canberra, Australia). For the 1RM assessment, athletes started shoulder width apart, with the barbell resting on the upper back approximately at the level of the shoulder process, with the knees and hips fully extended. Each athlete was instructed to descend until their thighs were parallel to the ground and then ascend to an upright position at maximal velocity. The depth of the squat was assessed by two supervisors positioned laterally to the power rack. The initial external load was set at 20 kg, was progressively increased in 20-kg increments when the mean velocity was greater than 0.75 m s^−1^, and was increased in 10-kg increments when the mean velocity ranged from 0.75 to 0.50 m s^−1^. The test ended when the mean velocity was less than 0.50 m s^−1^. Two repetitions were performed when the mean velocity was greater than 0.75 m s^−1^, and one repetition was performed when the mean velocity was less than 0.75 m s^−1^ [24]. The rest period between sets was set to 3 min. Athletes received velocity performance feedback immediately after completing each repetition to encourage them to perform all repetitions at maximal velocity. The MV collected under all loading conditions was modeled by a linear regression model, and the squat 1RM was estimated based on the load corresponding to an MV of 0.33 m s^−1^ [25]. The GymAware has been widely utilized in multiple studies as the gold standard for validating other velocity monitoring devices [24].

### 2.8. Statistical Analysis

All statistical analyses were conducted using SPSS software (version 25.0; SPSS, Inc., Chicago, IL, USA). The results were presented as means with their corresponding standard deviations (±SD). Statistical significance was set at *p* < 0.05. The normality of data distribution was checked using Shapiro–Wilk tests. The two-way mixed ANOVA of repeated measures (3 [FSQ, LSQ, and TRAD] × 2 [pre and post]) was used to investigate the effects of each training program on the 5–0–5, CMJ, broad jump, and 1RM back squat performance. When a significant main effect or interaction was found, the post-hoc tests with Bonferroni correction were used to analyze the pair-wise comparisons. The standardized effect sizes were used to express the magnitude of mean differences. Qualitative descriptors for Hedges’ g were employed to interpret the thresholds, with values of ≤0.20 indicating a “small” effect size, 0.21–0.79 indicating a “medium” effect size, and >0.80 indicating a “large” effect size.

## 3. Results

Jumping Performance

Table 1 shows the jumping performance variables and the results of the ANOVA. From all the considered CMJ variables, only the main effect of time for CMJ height reached significance (Table 1). The post-hoc comparison indicated a significant increase from pre-to-post-intervention in the LSQ (*p* = 0.002; ES = 0.58) and FSQ (*p* = 0.001; ES = 0.8) groups, but not in the TRAD (*p* = 0.676; ES = 0.07) group. In the case of broad jump length, a significant main effect of time was found (Table 1). The post-hoc comparison indicated a significant increase from pre- to post-intervention (*p* = 0.001; ES = 0.36).

Table 2 shows the 5–0–5 COD test time, 1RM back squat pre- to post-intervention, each intervention data, and the results of the ANOVA. A significant main effect of time for 5–0–5 COD test time on the turn with the dominant leg and 1RM back squat was observed. The post-hoc comparisons indicated a significant decrease from pre-to-post-intervention in the 5–0–5 COD test time on the turn with the dominant leg (*p* = 0.038; ES = 0.49) and a significant increase in 1RM back squats (*p* = 0.001; ES = 0.4).

## 4. Discussion

The aim of this study was to compare the effects of a 4-week resistance training program with squat and split-squat (in forward and lateral direction) exercises performed on a flywheel device or in a traditional manner on back squat 1RM, 5–0–5 COD test time, CMJ performance, and horizontal jump length in soccer players. The main finding of this experiment was that all training groups (TRAD, FSQ, and LSQ) significantly improved horizontal jump length, 5–0–5 COD time with a turn on the dominant limb, and 1RM back squats. In turn, both flywheel-resistance training groups (FSQ and LSQ) significantly improved their CMJ height, with no effect in the TRAD training group.

All training programs significantly improved different athletic performance measures, such as COD, jumping, and maximal free-weight back squat strength. However, interestingly, the improvements slightly differed between groups. Performing squats and split squats (regardless of whether forward or lateral) on the flywheel device contributed to significant gains in CMJ jump height after 4 weeks of bi-weekly flywheel training. This is in line with a recent study by Asencio et al. [26], which demonstrated that 4 weeks of bi-weekly flywheel training significantly improved CMJ height in soccer players. The authors compared horizontal (lunge and split squat), vertical (squat and split squat), and mixed-directed (combination of mentioned exercises) flywheel lower-body exercises. Surprisingly, in spite of different conditions, each group significantly improved CMJ height. Similarly, in the current study, the improvements in CMJ height in both FSQ and LSQ groups were not significantly different from each other. However, no such enhancement was reported in the TRAD group. Considering past research, the current study confirms that flywheel training can effectively increase CMJ height [17,18,26]. Yet, to the best of the authors’ knowledge, limited research has compared the effects of a flywheel and free-weight resistance training in soccer players [17,18]. Somewhat contradictory results were reported by Coratella et al. [17] and Sagelv et al. [18], who found a significant and similar increase after the flywheel and free-weight training program. However, the procedures of both aforementioned studies included high intensities >80% 1RM in free-weight resistance training programs, whereas, in the current study, the intensity was set to 60% 1RM during the whole training intervention. In turn, the inertia load of 0.10kg·m^2^ used was similar to that in a study by Coratella et al. [17], which is recognized as suitable to achieve eccentric overload [27]. Since resistance-training intensity is thought to be an important stimulus for neuromuscular adaptations [28], it is possible that the intensity used in the current study was sufficient and influenced the results significantly. Moreover, it is important to underline that no significant changes in the jump strategy assessed by time to take off and counter-movement depth in all examined groups were observed. Therefore, a significant increase in CMJ height indicates that athletes improved their ability to generate concentric forces, leading to greater CMJ heights. From these findings, it could be concluded that bilateral squats, as well as forward and lateral split squats, performed on a flywheel device were effective in evoking a positive adaptation in CMJ height in soccer players.

Surprisingly, in terms of the horizontal jump length, 1RM back squat, and 5–0–5 COD time with dominant limb turn, in all groups, a positive training effect was reported, with no significant inter-group differences. Although better results in the 5–0–5 COD tests were expected in the LSQ group than others, all groups similarly reduced 5–0–5 COD time, but only with the dominant leg turn, presumably because the current study only examined the 180-degree COD test. Therefore, it is possible that the use of a different test, e.g., with a change of 90 degrees, which more closely reflects lateral split squats, would result in a significant difference between the groups. Converging evidence was found by Nuñez et al. [29], which showed that 6 weeks of lateral split squats on a flywheel device led to significant improvements in 90-degree COD performance (time and COD deficit, for both dominant and non-dominant leg turns), but not in the COD with a 180-degree turn. Therefore, albeit speculative, in the procedure of the present study, an exercise of a greater extent matching 90-degree COD would be required. Moreover, it is interesting that the 5–0–5 COD time was reduced only during the change with the dominant leg, but not with the non-dominant one. Unfortunately, we did not control this during training. However, studies by Gonzalo–Skok et al. [20] indicate that the long-term adaptation to flywheel resistance training may differ depending on the training session starting with the weaker or stronger leg.

In reference to horizontal jump length, statistically significant and comparable improvements were achieved in all studied groups, likely suggesting the importance of the specificity of force application in inducing desirable training adaptations [30,31]. In this sense, despite the squats and split squats being performed on a flywheel device, there was still a vertical plane movement, as during free weights. Therefore, it may not transfer as efficiently to horizontal movements as in the case of the CMJ height. Accordingly, all groups performed hip-thrust exercises (at the same intensity and volume), which appear to be better transferred to tasks requiring a powerful hip extension, such as a horizontal jump than a vertical one [32]. This could explain the significant improvements after the cessation of the whole training program.

Positive training effects have been observed for the 1RM back squat as well, across all groups, with no statistically significant inter-group differences. Notwithstanding, the results of previous studies seem to indicate a slight superiority of free-weight training in 1RM back squat gains. For example, the study by Sagelv et al. [18] showed a significant increase in 1RM back squat performance after flywheel resistance training, but a significantly greater effect was obtained following free-weight training. While Coratella et al. [17] determined flywheel and free-weight training to be equally effective in augmenting the 1RM back squat, a larger effect size was found in the group performing free-weight training (effect size: 0.4 versus 0.73). Although there were no significant differences between the groups in the 1RM back squat gains in the current study, the FSQ group reported the largest magnitude of improvements (by ~10.8%, effect size: 0.66), while those observed after LSQ (to ~3.6%, effect size: 0.23) and TRAD were slightly smaller (to ~4.2%, effect size: 0.25). It seems that this fact can again be explained by the force specificity principle and the moderate intensity in the TRAD group. A slightly lower increase in the TRAD group seems to be the result of the applied intensity, which could have been a suboptimal stimulus for maximum back squat strength development. In turn, the only factor differentiating LSQ from the FSQ group was the manner in which split squats were performed. Hence, it seems that lateral split squats are not equivalently effective compared to forward ones in 1RM back squat improvement. In addition, it is worth noting that the duration of this study was shorter compared to the aforementioned studies (4 versus 6–8 weeks). It is possible that if the intervention lasted longer than 4 weeks, the observed changes in performance would reach the level of statistical significance. Therefore, further studies are required.

Based on the results of this study, flywheel resistance training appears to be more suitable for soccer players seeking to increase CMJ height. The increased emphasis on explosive power and vertical jump performance in flywheel training could be especially advantageous for soccer players during activities such as heading and leaping for high balls. The decision between free-weight and flywheel resistance training for soccer players should ultimately be based on individual requirements, training objectives, and available resources. Combining the versatility of free weights with the unique training stimulus provided by flywheel devices could be advantageous for enhancing overall soccer performance.

The results of this study should be considered in light of the following limitations. One of the main limitations was the relatively small sample size in each training group. Due to resource constraints and the specificity of our study population, recruiting a larger number of participants proved challenging. Moreover, although our participants had experience with resistance training and familiarization sessions were conducted, it should be kept in mind that they had no previous experience with the use of flywheel devices or other eccentric overload-resistance training methods. Additionally, despite the training volume being high, the training programs lasted only 4 weeks. Therefore, some of the differential effects of the training programs might have been manifested if the intervention had been longer. Furthermore, the training intensity and volume were not progressively increased in course of the study. In addition, of note is that the inertia applied was the same for all participants. Another limitation is that we did not make a comprehensive measurement of COD performance tests across different angles of direction change. Future studies with larger sample sizes and a broader range of training variations, including long-term interventions, should focus on the optimization of training variables. Additionally, comparing flywheel resistance training with other resistance-training methods and conducting mechanistic evaluations would further enhance the understanding of the overall benefits and applications of this training modality.

## 5. Conclusions

The results of this study showed that both free-weight and flywheel resistance training performed in-season contributed to significant improvement in 1RM back squat, horizontal jump performance, and 5–0–5 COD test time, while flywheel resistance training might be superior in CMJ height enhancement in soccer players. Therefore, a short and intensive block of the flywheel, as well as free-weight resistance training in-season, can be an effective training solution to improve a variety of power-based motor abilities.

## Figures and Tables

**Table 1:** Change in jumping performance.

Group	Pre (95%CI)	Post (95%CI)	ANOVA		
			Between Group Effect	Group × Time Interaction	Main Effect of Time
Counter-movement Jump Height [cm]					
FSQ	33.5 ± 2.9 (31.0 to 35.9)	35.9 ± 2.8 * (33.1 to 38.6)	F = 0.140; *p* = 0.87; η^2^ = 0.013	F = 3.543; *p* = 0.047; η^2^ = 0.252	F = 20.239; *p* < 0.001; η^2^ = 0.491
LSQ	34.1 ± 3.8 (31.7 to 36.6)	36.5 ± 4 * (33.8 to 39.3)			
TRAD	34.3 ± 3.2 (31.9 to 36.8)	34.6 ± 4.3 (31.8 to 37.3)			
Counter-movement Jump Relative Peak Power [W/kg]					
FSQ	50.6 ± 3.3 (46.3 to 54.9)	52.8 ± 3.0 (48.9 to 56.8)	F = 0.176; *p* = 0.840; η^2^ = 0.016	F = 0.319; *p* = 0.730; η^2^ = 0.029	F = 0.046; *p* = 0.832; η^2^ = 0.002
LSQ	52.1 ± 6.8 (47.8 to 56.4)	52.7 ± 7.4 (48.7 to 56.6)			
TRAD	51.9 ± 6.8 (47.6 to 56.2)	51.1 ± 4.9 (47.1 to 55.0)			
Counter-movement Jump Peak Velocity [m/s]					
FSQ	2.65 ± 0.16 (2.54 to 2.77)	2.67 ± 0.08 (2.58 to 2.77)	F = 0.176; *p* = 0.840; η^2^ = 0.016	F = 0.319; *p* = 0.730; η^2^ = 0.029	F = 0.046; *p* = 0.832; η^2^ = 0.002
LSQ	2.69 ± 0.17 (2.58 to 2.81)	2.71 ± 0.15 (2.60 to 2.80)			
TRAD	2.70 ± 0.14 (2.58 to 2.81)	2.68 ± 0.14 (2.59 to 2.78)			
Counter-movement Jump Time to Take Off [ms]					
FSQ	769 ± 174 (671 to 866)	810 ± 130 (716 to 904)	F = 0.388; *p* = 0.683; η^2^ = 0.036	F = 0.004; *p* = 0.996; η^2^ = 0.000	F = 1.820; *p* = 0.192; η^2^ = 0.080
LSQ	751 ± 90 (653 to 848)	786 ± 111 (692 to 880)			
TRAD	722 ± 121 (624 to 820)	759 ± 141 (664 to 853)			
Counter-movement Jump Depth [cm]					
FSQ	29.3 ± 7.8 (25.3 to 33.3)	30.1 ± 5.6 (26.4 to 33.7)	F = 0.183; *p* = 0.834; η^2^ = 0.017	F = 0.183; *p* = 0.834; η^2^ = 0.017	F = 2.520; *p* = 0.127; η^2^ = 0.107
LSQ	26.4 ± 5 (22.4 to 30.4)	27.4 ± 5.4 (23.7 to 31.0)			
TRAD	29.6 ± 1.7 (25.6 to 33.6)	31.4 ± 3.6 (27.8 to 35.1)			
Broad-Jump Length [m]					
FSQ	2.38 ± 0.08 (2.25 to 2.52)	2.40 ± 0.13 * (2.29 to 2.52)	F = 0.038; *p* = 0.962; η^2^ = 0.004	F = 1.619; *p* = 0.222; η^2^ = 0.134	F = 4.713; *p* = 0.042; η^2^ = 0.183
LSQ	2.33 ± 0.15 (2.20 to 2.47)	2.47 ± 0.09 * (2.36 to 2.59)			
TRAD	2.37 ± 0.27 (2.23 to 2.50)	2.40 ± 0.22 * (2.28 to 2.51)			

Data are presented as mean ± SD and 95% confidence interval (95% CI); * significant difference in comparison to pre-intervention within the condition; ANOVA—analysis of variance; FSQ—forward split squat flywheel training group; LSQ—lateral split squat flywheel training group; TRAD—traditional resistance training group; CMJ—counter-movement jump group.

**Table 2:** Changes in 5–0–5 change of direction test time and one-repetition maximum back squat.

Group	Pre (95%CI)	Post (95%CI)	ANOVA		
			Between Group Effect	Group × Time Interaction	Main Effect of Time
Turn with Dominant Leg [s]					
FSQ	2.31 ± 0.08 (2.24 to 2.38)	2.28 ± 0.08 * (2.23 to 2.33)	F = 0.423; *p* = 0.66; η^2^ = 0.039	F = 0.083; *p* = 0.921; η^2^ = 0.008	F = 4.881; *p* = 0.038; η^2^ = 0.189
LSQ	2.31 ± 0.08 (2.24 to 2.38)	2.26 ± 0.04 * (2.21 to 2.31)			
TRAD	2.33 ± 0.12 (2.26 to 2.40)	2.30 ± 0.09 * (2.25 to 2.36)			
Turn with non-dominant Leg [s]					
FSQ	2.35 ± 0.06 (2.30 to 2.39)	2.33 ± 0.09 (2.27 to 2.38)	F = 1.983; *p* = 0.163; η^2^ = 0.159	F = 0.474; *p* = 0.629; η^2^ = 0.043	F = 1.299; *p* = 0.267; η^2^ = 0.058
LSQ	2.31 ± 0.08 (2.26 to 2.36)	2.29 ± 0.08 (2.23 to 2.34)			
TRAD	2.36 ± 0.06 (2.31 to 2.41)	2.37 ± 0.06 (2.31 to 2.42)			
1RM Back Squat [kg]					
FSQ	120 ± 16 (107 to 132)	133 ± 21 * (119 to 147)	F = 1.470; *p* = 0.253; η^2^ = 0.123	F = 2.193; *p* = 0.136; η^2^ = 0.173	F = 15.448; *p* = 0.001; η^2^ = 0.424
LSQ	110 ± 16 (97 to 122)	114 ± 17 * (100 to 128)			
TRAD	120 ± 19 (107 to 132)	125 ± 19 * (111 to 139)			

Data are presented as mean ± SD and 95% confidence interval (95% CI); * significant difference in comparison to pre-intervention within the condition; ANOVA—analysis of variance; FSQ—forward split squat flywheel training group; LSQ—lateral split squat flywheel training group; TRAD—traditional resistance training group.

## Data Availability

Data is available on request from the corresponding author.
